# SuperPred 3.0: drug classification and target prediction—a machine learning approach

**DOI:** 10.1093/nar/gkac297

**Published:** 2022-05-07

**Authors:** Kathleen Gallo, Andrean Goede, Robert Preissner, Bjoern-Oliver Gohlke

**Affiliations:** Charité - Universitätsmedizin Berlin, Institute of Physiology and Science IT, Corporate Member of Freie Universität Berlin, Berlin Institute of Health, Humboldt-Universität zu Berlin, 10117 Berlin, Germany; Charité - Universitätsmedizin Berlin, Institute of Physiology and Science IT, Corporate Member of Freie Universität Berlin, Berlin Institute of Health, Humboldt-Universität zu Berlin, 10117 Berlin, Germany; Charité - Universitätsmedizin Berlin, Institute of Physiology and Science IT, Corporate Member of Freie Universität Berlin, Berlin Institute of Health, Humboldt-Universität zu Berlin, 10117 Berlin, Germany; Charité - Universitätsmedizin Berlin, Institute of Physiology and Science IT, Corporate Member of Freie Universität Berlin, Berlin Institute of Health, Humboldt-Universität zu Berlin, 10117 Berlin, Germany

## Abstract

Since the last published update in 2014, the SuperPred webserver has been continuously developed to offer state-of-the-art models for drug classification according to ATC classes and target prediction. For the first time, a thoroughly filtered ATC dataset, that is suitable for accurate predictions, is provided along with detailed information on the achieved predictions. This aims to overcome the challenges in comparing different published prediction methods, since performance can vary greatly depending on the training dataset used. Additionally, both ATC and target prediction have been reworked and are now based on machine learning models instead of overall structural similarity, stressing the importance of functional groups for the mechanism of action of small molecule substances. Additionally, the dataset for the target prediction has been extensively filtered and is no longer only based on confirmed binders but also includes non-binding substances to reduce false positives. Using these methods, accuracy for the ATC prediction could be increased by almost 5% to 80.5% compared to the previous version, and additionally the scoring function now offers values which are easily assessable at first glance. SuperPred 3.0 is publicly available without the need for registration at: https://prediction.charite.de/index.php.

## Graphical Abstract

**Graphical Abstract:**
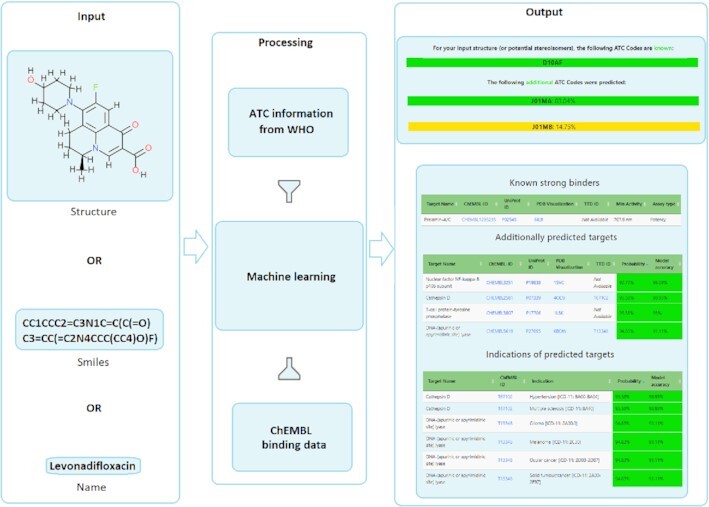
SuperPred 3.0: drug classification and target prediction—a machine learning approach.

## INTRODUCTION

Since its first publication in 1976, the Anatomical Therapeutic Chemical (ATC) classification system of the World Health Organization (WHO) is still the prevalently used classification system for approved drugs. Continuously updated to account for new findings, it classifies drugs according to their anatomical, therapeutic, pharmacological and chemical properties. It is built strictly hierarchically, with level 5 representing singular drugs and levels 1–4 increasingly larger groups of drugs with similar properties (1). Therefore, predicting the ATC group of an unknown substance can give insights into its medical properties and be used to assess drug candidates. Different methods have been proposed over the years to predict ATC classes, for instance supervised learning techniques for lower levels (2), with higher levels being predicted by combining the level 1 prediction with external sources such as ChEMBL (3) or STITCH (4). Other prediction methods are based on multi-label network inference (5–8) or deep learning in the form of convolutional neural networks (9). Similarly important when assessing properties of drug candidates are their interactions with human protein targets. Binding of a structure to unintended off-targets can potentially cause dangerous side effects (10–11) and therefore not only renders a substance unsuitable for approval but also offers chances for new therapeutic application areas, even for already existing drugs (12–13). Therefore, a wide variety of methods for the prediction of protein targets for unknown chemical structures have been developed, including network-based approaches, machine learning models, molecular docking and ligand-based *in silico* predictions (14).

However, only few webservers exist that offer ATC or target prediction services. In the last decade, only one other webserver for ATC prediction was published (8), which is currently not reachable. Similarly, available servers for target prediction are based on molecular docking (15–16) and therefore need a target protein in addition to the chemical structure of interest. Promiscuous 3.0 aims to overcome this limitation by providing a freely available webserver that offers both ATC and target prediction services for user-provided molecular structures, with the possibility to compare input structures simultaneously to all available targets and obtain prediction scores immediately.

## MATERIALS AND METHODS

### Data filtering for ATC prediction

To obtain a dataset suitable for the prediction of ATC codes for unknown chemical structures, a number of filtering steps needed to be applied. The initial dataset was obtained from the WHO website (https://www.whocc.no/) and contained 5067 distinct ATC codes that were spread over 795 different level 4 groups. In multiple stages, the dataset was polished to contain a remaining number of 1552 ATC codes over 233 predictable level 4 groups, satisfying the following requirements:

1. All ATC codes starting with the letter ‘V’ were removed from the dataset, as they belong to the group ‘various’, which can contain many different types of drugs and therefore a broad variety of potentially unrelated structures.
2. To enhance the unambiguity of the dataset, all singular ATC codes that belong to a combination of two or more different drugs were removed.
3. As another step to ensure the unambiguity of the ATC groups in the prediction dataset., structures that were associated with different ATC codes had all their respective codes removed from the dataset.
4. Since the prediction is based on molecular structure, only ATC codes that were assigned to structures and can therefore be translated into a SMILES string were kept. This removes ATC codes associated with plants, materials, proteins and similar substances from the prediction dataset.
5. Salts were removed as well. Especially for the validation, inclusion of salts would lead to an overestimation of the prediction performance, since the same chemical structure could be present multiple times in the training dataset, with only the secondary atom/a very small chemical substructure being different between the substances.

After applying the described filtering steps, the remaining ATC codes were grouped according to their respective level 4 codes. Furthermore, all groups that consisted of only one remaining structure were removed as well, since their inclusion would likely lead to extreme overfitting. Lastly, all groups ending on the letter ‘X’ were excluded, since they are established for newly added substances that are not clearly belonging to an existing level 4 group and therefore classified as ‘other’ structures in the respective level 3 groups.

### Data filtering for target prediction

The dataset for the target prediction was obtained from the ChEMBL database (3), using version 29. As for the ATC code prediction, the binding dataset needed to be filtered first, which was done similarly as in Peón *et al.* (17). Only assays for the organism of ‘Homo sapiens’ were included, and the assay type had to be either type B (binding) or type F (functional). Furthermore, the associated assay confidence level had to at least 7. The denoted activity types were required to be IC50, EC50, Ki, Kd or Potency, with a standard unit of nanometer (nm), with values of 0 nm additionally excluded.

Using the remaining binding assay data, we defined two sets of associated structures for each target: strong binders and non-binders. For strong binders, at least one activity value for the specified target structure had to be 1000 nm or less, with the published relation of ‘ = ’ or ‘<’. Similarly, non-binders were defined as substances with activity values of 30 000 nm or more and a relation of ‘ = ’ or ‘>’. Additionally, potential ambiguity from the varying assay data was removed by requiring that for strong binders no further assay exists that defines it as a non-binder and vice versa. Additionally, in this way binding data with the data validity comment ‘Outside typical range’ were excluded from both sets.

These filtering steps resulted in 500 979 unique trusted relations between 365 719 strong binding substances and 2353 targets. About 691 of these have assigned at least 20 binders and nonbinders, respectively, and therefore enough associated data to be part of the prediction pipeline.

### Machine learning methodology

As training data for the machine learning models, the molecular structures with known ATC codes and target relations were used in the form of molecular fingerprints, which were derived from their corresponding SMILES representation.

For both ATC and target prediction, a number of different machine learning models were tested and evaluated regarding their performance. This included logistic regression, linear discriminant analysis, *k*-nearest neighbors, decision tree, support vector machines, gaussian naïve bayes and random forests. In addition to this, a combination of varying parameters was tested regarding length and (if applicable) radiuses for the molecular fingerprints. Furthermore, since the size of level 4 groups greatly varied, with the largest groups containing 52 structures and smaller groups only 2–3, random oversampling was applied to the training dataset, meaning a structure from the minority class was randomly chosen to be re-inserted into the dataset, until all classes were of the same size.

Of the tested models, logistic regression using Morgan fingerprints of length 2048 and radius 2 achieved the highest accuracy (evaluated via leave-one-out cross-validation), classifying 70.1% of level 4 ATC codes correctly. Prediction of the ATC code for an unknown molecular structure was performed using a multi-class prediction model, which does not evaluate the probability for each of the 233 predictable ATC classes separately but instead works with the assumption that each structure belongs to exactly one ATC group and therefore distributes a total value of 100% between the different classes. This corresponds to the filtered training dataset, where ambiguous ATC codes were previously removed. Still, structures associated with multiple ATC codes can be recognized via the distribution of the resulting probabilities, when multiple high scoring ATC codes are predicted.

**Figure 1.**
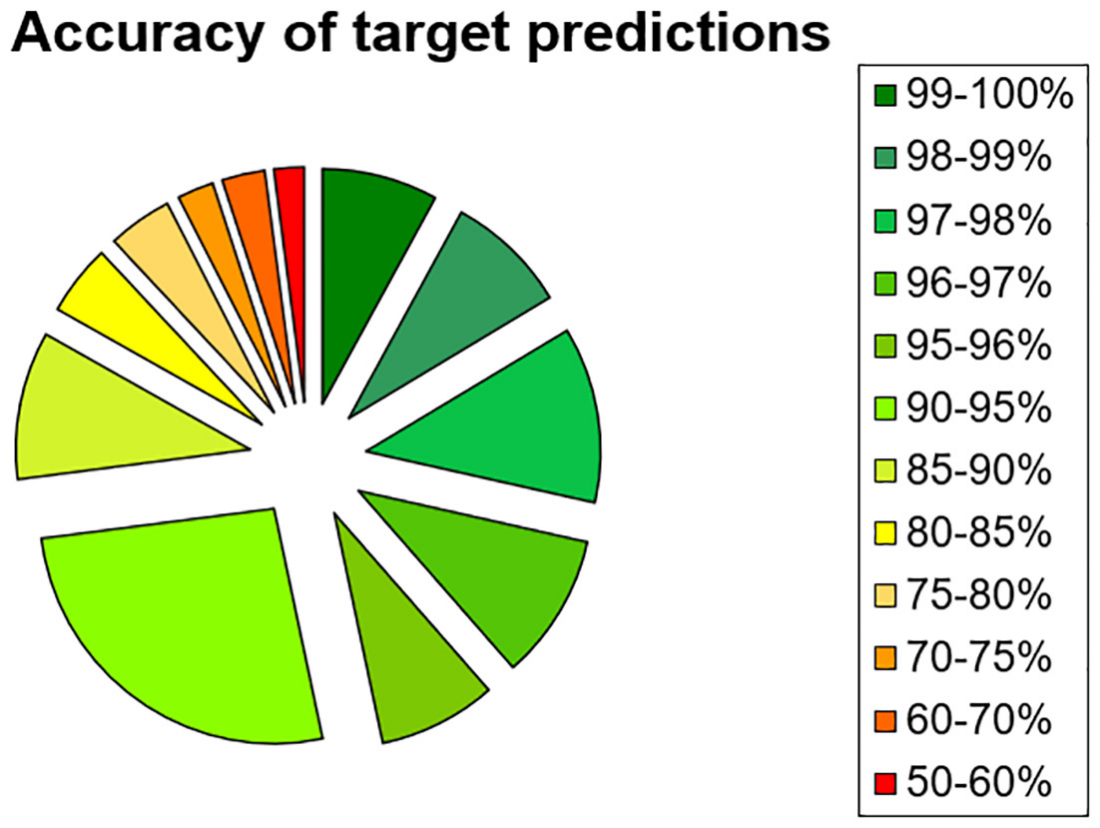
Overview of the accuracies for the different target prediction models, evaluated via 10-fold cross-validation.

For the target prediction, one machine learning model was trained for each of the predictable targets. Accuracy of the models was evaluated using 10-fold cross-validation, with 82% of the target models achieving at least 85% accuracy and only 5% scoring worse than 70% accuracy (Figure 1). Since the performance of the machine learning models varies between targets, two different scores are reported as results: the probability that the input structure interacts with the target in question as determined by the corresponding machine learning model, and the overall accuracy of the respective model.

### Assigning of indications

As an additional feature, possible relevant indications for predicted targets were extracted from the Therapeutics Target Database (TTD, 18). Each of the included predictable targets was mapped to targets in the TTD database and their associated indications are displayed in a separate table, if the respective target is predicted to be active for a structure of interest. If multiple indications are assigned to a single target, each indication is displayed in a separate row to facilitate searching the table for indications that are shared between multiple different predicted targets.

## THE SUPERPRED 3.0 PLATFORM

### Server architecture

Data for the webserver is stored in a relational MySQL database, hosted on the Charité IT system. For the handling of chemical information in the database, as well as for the preprocessing of the training data for the machine learning models, the Python package RDKit (http://www.rdkit.org/) and ChemAxon (https://chemaxon.com/) software were used. The webserver back-end consists of a lab-based LAMP (Linux/Apache/MySQL/PHP) server, using PHP as back-end language. The connection to the underlying database is established through a MySQL interface, and front-end data delivery via a mixture of Html form submission responses and AJAX requests. Website functionalities are implemented using JavaScript and, in extension, its plugin jQuery (https://jquery.com/). Additionally, the CSS framework Bootstrap 4 (https://getbootstrap.com/) is used. Tables on the website are created using the jQuery plugin DataTables (https://datatables.net/) and its absolute sorting extension (https://datatables.net/plug-ins/sorting/absolute). For the chemistry interface, the JavaScript library ChemDoodle Web components (https://web.chemdoodle.com/) was used.

For further security improvements, we activated Fail2Ban (https://www.fail2ban.org/wiki/index.php/Main_Page) as well as an App-Firewall. Fail2Ban inspects logs and reacts to suspicious signatures via IP-bans at the firewall. By this, it blocks automated mass-scan and route attack, and also reinforces other security systems by adding bans for perpetrators. In addition, we also implemented an App-Firewall, configured specifically to this application, that actively blocks many dangerous requests before they can happen. Here, both security systems are used in conjunction, by additionally configuring Fail2Ban to ban anyone violating App-Firewall rules. An app firewall protects from many threats, from cross-side scripting (CSS) to SQL-injections, session hijacking and more. By this, it could happen to users that they will be blocked by one of these security mechanisms, if they use the webserver in an incorrect way. Should this happen, they will remain blocked for a short period, where the site will not load for this IP-address.

The usage of a JavaScript-capable browser is essential and the server was tested on the most recent versions of Google Chrome and Mozilla Firefox. No login or providing of email address is needed to access all functionalities of the webserver.

### Data input

Both ATC and target prediction require a molecular structure as input parameters, using the same input mechanisms. Structures can be uploaded by a number of ways in the ChemDoodle web interface. The simplest possibility is entering the PubChem (19) name of a structure of interest, but SMILES strings and molecular structure files can be entered/uploaded as well. Additionally, drawing tools are provided, either to draw a molecular structure from scratch or to modify an uploaded structure. When satisfied with the displayed structure, calculations can be started using a simple button-press.

### Output data

Calculations should take only a couple seconds, and results are displayed immediately on the webpage for both ATC and target prediction.

Prediction of the ATC code for an input structure displays all known ATC codes, in case of the structure already being included in the SuperPred 3.0 database. Known ATC codes are reported without a probability score, to further differentiate them from the predicted ATC groups. Depending on the probability score, newly found ATC codes are reported either in green, yellow or red color, and structures with multiple potential ATC codes are highlighted in particular. It is possible that both known and newly found, potential ATC codes are reported.

Similarly, using the target prediction for structures that are included in the database, both known and newly predicted targets can be reported. For this purpose, only targets that classify as ‘strong binders’ (as described in the data filtering for the target prediction) are considered known targets and displayed with their corresponding assay binding data. Predicted targets are reported in form of a table, providing the formerly described two accuracy scores as well as information and visualization links. Additionally, associated indications of predicted targets are displayed in a separate table, according the TTD mapping. Targets with multiple indications are displayed with separate rows for each indication, so the table is easily searchable by (shared between different targets) indications.

### Comparison to other methods/servers

A challenge when comparing performances between different methods is the inexistence of a standard ATC prediction dataset, on which accuracies of different models can be evaluated. Furthermore, multiple different metrics exist for the assessment of performance, and lastly not all webservers aim to predict the same level of ATC groups. Therefore, accuracies are compared both for level 1 and level 4 ATC prediction (even though the models used in SuperPred 3.0 were optimized only for level 4 prediction), and for publications where a different measure of performance was used, the analogous performance metric was calculated. Additionally, to ensure comparability to the previous version of SuperPred, performance was evaluated on the previously used dataset as well.

Compared to other recent publications, SuperPred 3.0 performs best, both in level 1 and level 4 prediction. Additionally, performance on the legacy dataset could be increased by almost 5%, compared to SuperPred 2.0 (Table 1). Only one of the publications also offers their method in form of a webserver, which is unfortunately currently not reachable, so that, to the best of our knowledge, SuperPred 3.0 is the only freely available webserver offering ATC code prediction.

**Table 1.** Comparison of the accuracy of different ATC prediction servers. * = accuracy for expected ATC code reported as first hit evaluated from AUC figure, ** = using SuperPred 2.0 dataset, *** = using SuperPred 3.0 dataset

Publication	Level 1 accuracy [%]	Level 4 accuracy [%]	Webserver
Olson, 2017	73.7	41.2	No
Cheng, 2017	67.1	-	No
Lumini, 2018	77.8	-	No
Wang, 2019	79.5	-	No
Peng, 2021	-	33*	Not reachable
SuperPred 2.0	80.9	75.1	-
SuperPred 3.0	87.9**/82.3***	80.5**/70.1***	Yes

Among the webservers doing target prediction, comparable servers are either currently not reachable (20–21), not displaying a result (22) or using a docking approach (15–16), which requires the desired target to be provided additionally. In contrast, SuperPred 3.0 allows the comparison of an input structure to all available targets immediately.

### Example case

**Figure 2.**
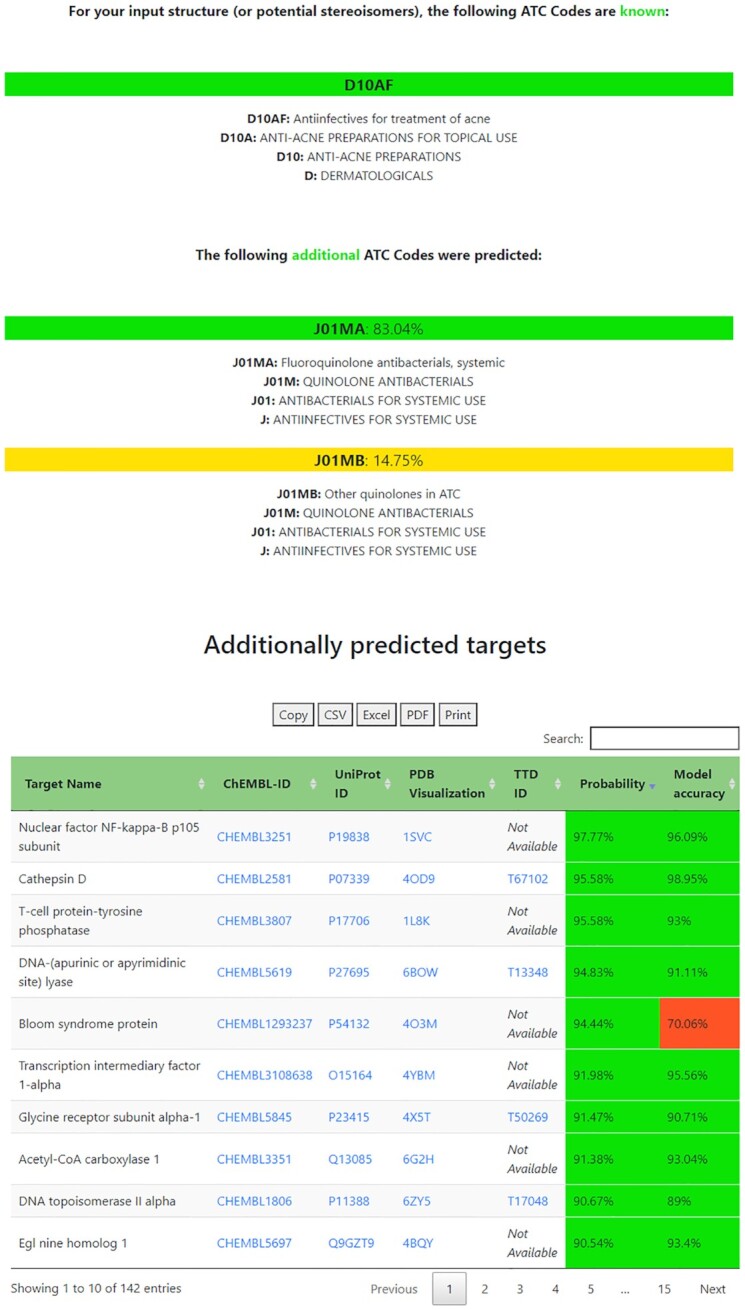
Computational output for ATC and target prediction of the substance levonadifloxacin.

To demonstrate the functionalities of the webserver, the newly approved drug levonadifloxacin was chosen, which was assigned with the ATC code J01MA24 in 2021. It is a stereoisomer of the previously approved drug nadifloxacin, more specifically an arginine salt of the active S(-)isomer (23). With the ATC code being assigned so recently, it was not included in the training data for the prediction dataset and is therefore unknown to the database. In contrast, nadifloxacins ATC code D10AF05, which has been established for a longer time, is included both in the database and the prediction training dataset. Therefore, performing an ATC prediction for levonadifloxacin first reports D10AF as an ATC code, that is known for a stereoisomer of the structure in question. Despite a stereoisomer being included in the training dataset, J01MA and J01MB are additionally predicted as potential ATC codes. These groups only differ in the last letter and contain structurally similar substances. Still, the actual ATC group J01MA is predicted with a likelihood of 83%, while J01MB only achieves a score of 15% (Figure 2).

Levonadifloxacin is an antibacterial agent that was approved for the treatment of acute bacterial skin and skin structure infections (24). Among its predicted targets is DNA topoisomerase II alpha, which is predicted to be associated with levonadifloxacin with a probability of over 90%, while the respective target prediction model was evaluated with an accuracy of 89% via 10-fold cross-validation (Figure 2). DNA topoisomerase II alpha is listed on TTD as a functionally relevant target for other drugs from the ATC group J01MA, such as ciprofloxacin, which also share antibacterial properties.

## CONCLUSIONS

The prediction method of SuperPred has been completely reworked and is now based on machine learning models instead of overall structural similarity. This allows for the accurate prediction of ATC groups, even in cases where only small parts of the respective structures, such as functional groups, are responsible for the therapeutic impact or metabolic processes and therefore the assignment to a specific ATC code. By these means, accuracy of the ATC prediction could be improved by more than five percent in comparison to the previous version of the webserver.

The prediction of (therapeutic) targets is no longer based only on active binders but also includes experimentally confirmed nonbinders, which were extracted from the ChEMBL database. Together with the machine learning methodology, this design enables a much more accurate assessment of structural groups that play a role in the protein binding process, in addition to the advantage that focusing on substructure features in contrast to overall structural similarity already offers. Furthermore, the previous scoring function was replaced with much more intuitive values, which are easily assessable on first glance.

Lastly, the inexistence of a commonly used ATC dataset complicates the comparison between different prediction approaches. Here, we made an effort to compile and accurately filter an ATC dataset which is both suited for the precise prediction of ATC codes, but at the same time downsamples all too similar structures, which leads to a more accurate assessment of the performance and simultaneously serves to avoid overfitting in unknown datasets. The filtered dataset is available in the supplementary material, including information about expected and predicted ATC codes.

## DATA AVAILABILITY

SuperPred 3.0 is publicly available and accessible without any need for registration at https://prediction.charite.de/index.php. Results are displayed immediately and directly on the website without the need to provide an e-mail address or similar means. The filtered dataset (including results from the performed leave-one-out cross-validation) is both available in the supplementary data and downloadable in the website FAQs.

## Supplementary Material

gkac297_Supplemental_Files
